# Symptoms and impacts of aromatic l-amino acid decarboxylase (AADC) deficiency among individuals with different levels of motor function

**DOI:** 10.1186/s13023-022-02274-0

**Published:** 2022-03-21

**Authors:** Kate Williams, Hanna Skrobanski, Katharina Buesch, Sarah Acaster

**Affiliations:** 1 Acaster Lloyd Consulting Ltd, London, UK; 2 PTC Therapeutics Switzerland GmbH, Steinhausen/Zug, Switzerland

## Abstract

**Background:**

Aromatic l-amino acid decarboxylase (AADC) deficiency is a rare neurological disorder associated with a range of symptoms and functional impairments. The aim of this study was to describe the experience of AADC deficiency across five different motor milestone health states.

**Methods:**

Qualitative interviews were conducted with caregivers of individuals with AADC deficiency in Italy, Spain, Portugal and the United States. An interview guide was developed with input from clinical experts and caregivers, and explored the symptoms and impacts of AADC deficiency. Interviews were conducted by telephone and were recorded and transcribed. Data were analysed using thematic analysis and the symptoms and impacts were compared across health states.

**Results:**

Fourteen caregivers took part, who provided care to 13 individuals with AADC deficiency aged 1–15 years. Six individuals were in the ‘no motor function’ health state, one in the ‘sitting unsupported’ health state, one in the ‘standing/stepping when fully supported’ health state and five in the ‘walking with minimal support’ health state. The results highlight a substantial impact of AADC deficiency, even among those who were able to walk with minimal support. Overall, those with better motor function also had better functional hand use, communication skills, ability to eat and perform other activities independently, and interact with their peers. The burden of caring was high across all health states, but caregivers of individuals in the walking health state were better able to participate in social and leisure activities.

**Conclusion:**

Individuals with higher levels of motor function had less severe symptoms and were better able to perform their daily, leisure and social activities. Treatments which improve motor function have the potential to improve other aspects of the lives of individuals with AADC deficiency and their caregivers.

## Background

Aromatic l-amino acid decarboxylase (AADC) deficiency is a rare, autosomal recessive neurometabolic disorder, with an estimated prevalence of between 1:64,000 and 1:90,000 births in the USA, 1:116,000 in the European Union, 1:162,000 in Japan and 1:32,000 in Taiwan [1–3]. It is associated with developmental delay, in addition to a wide range of other symptoms and functional issues, including hypotonia, oculogyric crises (upward deviation of the eyes lasting from seconds to hours [4]), mood and sleep disturbance, autonomic and gastrointestinal dysfunction, and eating difficulties [5–7]. Due to the broad spectrum of symptoms and functional issues associated with AADC deficiency, most individuals require life-long care [6].

There are currently no licensed treatments specifically for AADC deficiency [6, 7], so existing treatments aim to control symptoms and improve health-related quality of life (HRQoL). Clinical trials are currently evaluating the efficacy of gene therapies, which have the potential improve the motor function and cognitive development of individuals with AADC deficiency [8–11]. Exactly what improvement may mean to individuals living with AADC deficiency and their caregivers may be difficult to fully appreciate, as little is published about the patient and caregiver experience at different levels of functioning. Understanding the patient relevance and value that treatments may provide is critical for patient access to treatment. In many countries worldwide, access to treatment is determined by health technology assessment agencies (HTA) evaluation of treatment benefit, which includes the patient relevance of ‘benefits’ and the treatment impact on HRQoL. Typically this improvement is depicted in an economic model, with improvement or worsening in a condition (e.g., motor function differences in AADC deficiency) represented by different health states in the model. The HRQoL differences between health states are often represented by utility weights assigned to each health state. Utility values range from 1 (full health) to 0 (dead) with states worse than dead (negative values) also possible. In rare disease there can be a great deal of uncertainty around utility estimates, due to small sample sizes and heterogeneity of disease. Furthermore, the meaning of utility gain/difference is not very transparent to patients, caregivers and clinicians, who are likely to be the stakeholders making the treatment decisions.

Qualitative research generates rich data on what it means to live with a condition, by allowing participants, for example, patients or caregivers, to speak freely and describe their experience in their own words. Recent qualitative research has highlighted the extensive impact of AADC deficiency on individuals living with the condition [12]. This study aimed to extend this previous work by exploring the data stratified into five health states related to motor milestones based on the Peabody Developmental Motor Scales-2 (PDMS-2): (1) full head control, (2) sitting unsupported, (3) standing/stepping with support and (4) walking with minimal support, and (5) no motor function. Understanding the HRQoL of individuals in each of these health states qualitatively may help regulators better understand the impact of treatment from the patient and caregiver perspective and help shared decision making as treatments become available.

## Methods

Full details of the methods have been published previously [12], but are described briefly below.

### Design and participants

Qualitative interviews were conducted with caregivers of individuals with AADC deficiency in Italy, Portugal, Spain and the United States. The interviews were conducted with informal (unpaid) caregivers, as the individuals with AADC deficiency were too severely impaired to participate in an interview.

### Study materials

A background questionnaire was developed to collect socio-demographic information and information on the individual with AADC deficiency’s disease background and treatments. This included questions adapted from the PDMS-2 about the individual’s current level of motor function, which allowed them to be characterised into no motor function, head control, sitting without support, standing/stepping when fully supported and walking with minimal support health states (Table 1).

**Table 1:** Health state categorisation

Level of function	Health state
Unable to hold their head level for 8 seconds while rotating their head from side to side to follow a moving object	No motor function
Able to hold their head level for 8 seconds while rotating their head from side to side to follow a moving object	Head control
Can sit unsupported for 60 seconds	Sitting without support
Can take four alternating steps on the spot or forward, when supported by someone holding them around their body	Standing/stepping when fully supported
Can use alternating steps to walk 8 feet (2.5 m) with minimal support (holding their hand)	Walking with minimal support

A semi-structured interview guide was developed which comprised mainly of open-ended questions on the individual with AADC deficiency’s diagnosis and symptoms, as well as the impacts on their daily life and health-related quality of life, and the impacts on their caregiver.

### Ethics review and approval

This study was submitted for ethical review by the WIRB-Copernicus Group Independent Review Board and was granted an exemption (tracking number: #1-1327023-1).

### Recruitment and interviews

Participants were sent an information sheet about the study along with a background questionnaire to complete and return by email. The interviews in the United States were conducted in English by two study authors. The remaining interviews were conducted by trained interviewers in each study country in the local language. All interviews were conducted by telephone/videoconference between September and December 2020, and lasted around an hour. Interviews were recorded and transcribed, then translated into English for analysis by a specialist translation vendor.

### Analyses

Data from the background questionnaire were summarised using descriptive statistics. Participants were initially cateogorised into the different health states based on their responses to the adapted PDMS-2 questions in the background questionnaire. Where there were discrepancies between their questionnaire response and their qualitative data on motor function, a clinician assigned them to a health state based on their qualitative data. This clinician was a board-certified physician specialising in psychiatry and psychotherapy, with 2.5 years of experience treating individuals with AADC deficiency or other related care conditions.

Data from the interviews were analysed using thematic analysis in MAXQDA. Two researchers read all the transcripts and developed a coding framework based on the topics covered in the interview guide. One researcher then coded a sample of transcripts and these were reviewed by a second researcher and discrepancies were discussed. The coding framework was revised following this discussion and the remaining transcripts were coded. Additional data-driven amendments were made to the coding framework throughout the coding process. The codes were then grouped into themes to describe the experience of living with, or caring for someone with, AADC deficiency, which were subsequently compared across the different health states.

## Results

### Sample characteristics

Fourteen interviews were conducted with caregivers of individuals with AADC deficiency. Two caregivers were parents of the same individual. The caregiver characteristics are shown in Table 2.

**Table 2:** Caregiver characteristics (N = 14)

Characteristic	Mean (SD)
Age (years)	43.9 (6.9)

	N (%)
Ethnic background (N = 8)	
White	7 (87.5)
Hispanic/Latino	1 (12.5)
Education	
No formal qualifications	1 (7.1)
School	3 (21.4)
University degree or higher	10 (71.4)
Employment status	
Employed full-time	1 (7.1)
Employed part-time	1 (7.1)
Self-employed	2 (14.3)
Full-time homemaker/caregiver	9 (64.3)
Other	1 (7.1)
Country	
Italy	5 (35.7)
United States	7 (50.0)
Spain	1 (7.1)
Portugal	1 (7.1)
Relationship to individual with AADC deficiency	
Mother	10 (71.4)
Father	2 (14.3)
Brother	1 (7.1)
Aunt	1 (7.1)

The characteristics of the individuals with AADC deficiency are shown in Table 3. There were six individuals in the ‘no motor function’ health state (with data from seven caregivers), one in the ‘sitting unsupported’ health state, one in the ‘standing/stepping when fully supported’ health state and five in the ‘walking with minimal support’ health state. There were no participants in the ‘head control’ health state.

**Table 3:** Characteristics of individuals with AADC deficiency (N = 13)

Characteristic	Mean (SD)	Range
Age (years)		
When first symptoms noticed (months)	11.9 (11.9)	0–48
At diagnosis with AADC deficiency (months)	30.1 (23.0)	7–96
Current (years)	6.9 (4.7)	1–15

	N (%)
Sex	
Male	9 (64.3)
Female	5 (35.7)
Current health state	
No motor function	6 (46.2)
Head control	0 (0)
Sitting without support	1 (7.7)
Standing/stepping when fully supported	1 (7.7)
Walking with minimal support	5 (38.5)
Uses a feeding tube	5 (35.7)
Uses a ventilator	4 (28.6)

### Symptoms and impacts among those with no motor function (Fig. 1)

**Fig. 1:**
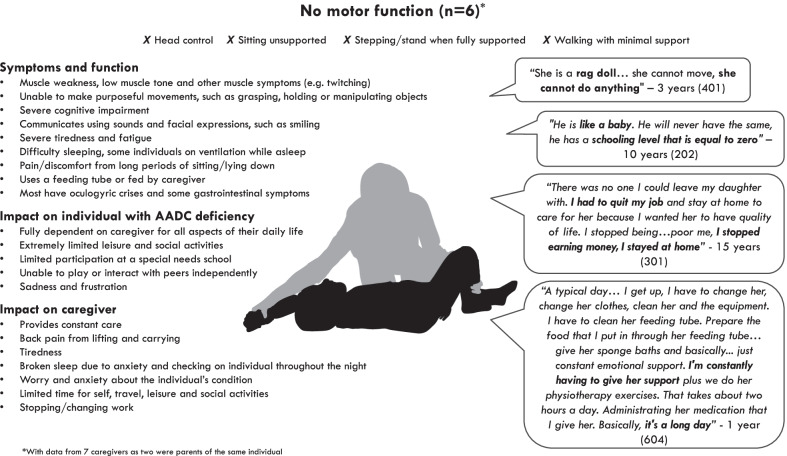
Overview of symptoms and impacts among those with no motor function

#### Symptoms and function

In line with their health state categorisation, those in the ‘no motor function’ health state were unable to hold their head up, sit unsupported, stand or walk. They also had limited upper limb function and most were unable to raise their arms above their head, reach for objects or grasp and manipulate objects.

*"He can’t hold onto things very well, at all really. He can push them and… he can push things around, but he can’t hold on" –* 1 year (participant 607)

Some caregivers described individuals as being “floppy” or like a “rag doll”. Other muscle and jointed-related symptoms included muscle spasms and twitches, stiffness and abnormal posture.

Cognitive function was also impacted in this health state. Some described how the individuals stopped developing and had the cognitive abilities of a baby or young child, unable to speak with words and sentences. Despite this, caregivers reported that the individuals had an awareness of their surroundings and were able to recognise familiar faces and sounds and use sounds and/or use facial expressions and eye movements to communicate.

*“You notice that she is aware of everything, that she knows people perfectly, that she recognises people, the physiotherapists, for example, she hears their voice, she knows perfectly who they are”* – 3 years (participant 401)

*"If I hold things up, I could see by her facial expressions... that’s what she’s wanting is her pacifier... I would say she can identify with objects. And, like, if I show her certain things, like a teddy bear, you know, she'll… she knows that's her teddy bear she'll smile"* - 1 year (participant 604)

Several caregivers reported that the individuals in this health state experienced episodes of oculogyric crises. These episodes were reported to affect the whole body, and included characteristics such as eyes rolling back in the head, staring upwards, stiffness, shivering, pain, and involuntary movements.

*“The first thing I notice is her stare. Her eyes seem empty, she seems to be in another world. Her eyes roll upward a lot. For instance, the iris comes forward and she “buries” it, I do not know how to explain it. And she chews her tongue a lot. She starts moving, the arms also start to tremble and she shivers. It is hard to control, it is difficult” -* 15 years (participant 301)

Others reported that they had epileptic seizures. These were described in a similar way to oculogyric crisis, and were reported to affect the whole body.

Some caregivers of individuals in this health state reported that they experienced gastrointestinal symptoms, including diarrhoea, nausea, retching and difficulty gaining weight. Four individuals were fed through a feeding tube because they did not have the muscle control to hold their head up, or to help manage some of their gastrointestinal symptoms. The remainder were able to eat by mouth but were fed by their caregivers. These individuals were reported to eat liquid or blended foods because they had difficulty swallowing and holding their head.

Several caregivers reported that the individuals in this health state experienced fatigue and tiredness because any activity would use a lot of energy.

*“I notice that when I go out with her, for her it is almost like running a marathon. She gets very tired”* - 15 years (participant 301)

*“She is super happy for half an hour there, stretching, moving, turning. Then, it seems like she runs out of battery a bit later, half an hour or earlier, it seems that she, that she ran out of battery"* – 3 years (participant 401)

However, despite this tiredness and need to sleep, several caregivers described how the individual would often struggle to fall asleep and some reported that they gave them melatonin to help with this. Two individuals were reported to need ventilation to help them breath at night and another also received ventilation when napping during the day.

Several caregivers reported that the individual experienced pain or discomfort, although they noted that it was difficult to know when the individual was in pain because of their limited communication abilities. Pain and discomfort were typically described as being a result of sitting or lying in the same position for long periods of time because they were unable to move.

#### Impact on the individual with AADC deficiency

All caregivers reported that the lives of individuals were severely limited and were unable to do anything without support. This included needing help with all aspects of self-care, including washing, dressing, changing and eating. Their limited hand use also made it challenging for them to play independently as they were unable to make purposeful movements.

*"A typical day for her… she can do nothing, the only thing that comforts her are the walks, and well, then we put her on the blanket, we play like a little bit, but all the movements she makes, we are the ones making them"* – 3 years (participant 401)

Because of these limitations, their main leisure activities were reported to involve walks in the park in a wheelchair and watching television. Some caregivers reported that the individuals went to school or nursery to give them an opportunity to be around other children and have social interaction. However, they were unable to participate in the same activities as other children and needed constant support.

*“He goes to school when possible, he cannot attend school every day like the normal children, he plays but also in this case, he plays because I make him play, I sit there with him, because he cannot even raise his arms alone”* – 8 years (participant 204)

Some caregivers reported that the individuals would sometimes become upset because of symptoms, such as reflux, or because they were tired. Others reported that the individual would become frustrated when they were unable to do something or because they were struggling to communicate their needs.

*“She does cry a lot. I think too because not being able to be verbal and communicate and things… I know most babies can't really communicate anyways but I feel… I can tell, like, you know, she gets frustrated and she wants to be able to do things”* - 1 year (participant 604)

#### Impact on caregivers

Caregivers reported providing constant care to individuals in this health state, including washing, dressing and feeding. They also reported spending a substantial amount of time managing the individual’s medical routine, including washing and changing their feeding tubes, helping them with their therapy sessions (e.g. physiotherapy) and taking them to medical appointments.

Several caregivers reported a physical impact of caring, including back pain as a result of having to lift and carry the individual and tiredness from their caring responsibilities.

*“Yes, cervical pain, back pain because anyhow he doesn’t weight a lot, twenty five kilograms but it’s not little, when I need to pull him up, I’m not old but I’m not twenty years old, I have back pain, cervical pain and when I reach the evening, I’m really tired” –* 10 years (participant 203)

Some reported that they had broken sleep because they would check on the individual during the night to make sure they were still breathing.

In addition to the physical impact, caregivers described the emotional burden of being a caregiver. Two individuals reported that they suffered from depression and others described the sadness they felt from watching the individual struggle or for the loss of a life they expected to have. Some mentioned feelings of guilt for not being able to give the individual what they needed, anxiety about the individual’s condition, and worry and fear for the future.

*“I’m anxious always, I think this is something that will die with me because anxiety doesn’t make me sleep at night, that doesn’t allow me to put my son in the other room, I’m anxious. I’m scared something could happen, I’m not ready to help him if something happens”* – 8 years (participant 204)

Most caregivers reported that they had very little time for themselves. For example, some described how they had very little time to get ready in the morning and often put off attending their own medical appointments.

*“Totally abandoned. I mean, you don't even to gynaecological check-ups, I talked about this with my husband yesterday, I mean, I have not even been to a check-up, I have not been to the dentist”* – 3 years (participant 401)

This lack of spare time meant that caregivers had very little time for social and leisure activities, or exercise. Similarly, several caregivers reported that they had needed to stop working or change jobs because of their caring responsibilities.

### Symptoms and impacts among those able to sit unsupported (Fig. 2)

**Fig. 2:**
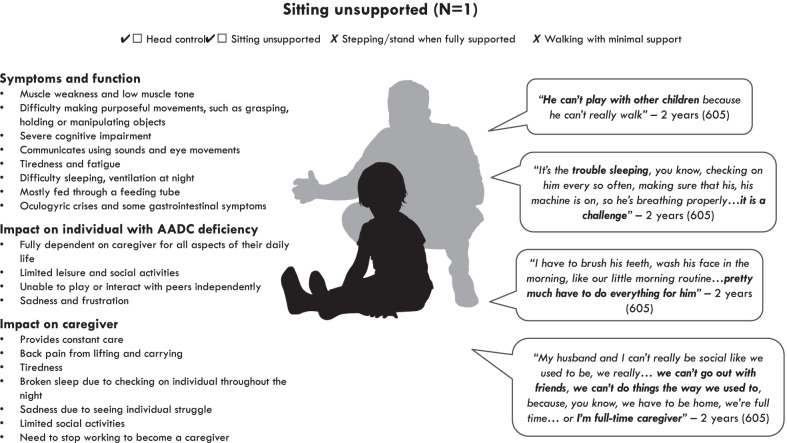
Overview of symptoms and impacts among those able to sit unsupported

#### Symptoms and function

The one individual in the ‘sitting’ health state was able to hold their head up and sit unsupported for 60 seconds, but could not stand or walk. Similar to those in the ‘no motor function’ health state, this individual experienced difficulty making purposeful movements and was unable to grasp, hold or manipulate objects.

*“He really can’t grab things, I try to give him things, we do have a couple of toys that have holes in them, so he can stick his fingers in there. So it looks like he’s holding it, but he’s really not"* – 2 years (participant 605)

The caregiver also reported that the individual had limitations with cognitive function, although they acknowledged that it was difficult to know because the individual was also unable to communicate verbally.

*“As far as memory, because he really can’t talk, it doesn’t… to me doesn’t seem like he has much of a memory right now”* – 2 years (participant 605)

The caregiver described how the individual made sounds to communicate rather than using words. The caregiver said that the individual would communicate non-verbally by looking in the direction of something they wanted.

Similar to those with no motor function, the caregiver of this individual reported that they experienced episodes of oculogyric crises, and seizures which would typically occur during the night.

This caregiver reported that the individual experienced some gastrointestinal symptoms, but these differed slightly from the individuals with no motor function. They were described as having trouble with digestion and constipation, which had resulted in the individual having a feeding tube fitted. The caregiver explained how these gastrointestinal symptoms were alleviated by adding water to their feeding tube. While most of the individual’s nutrition was provided through a feeding tube, the caregiver would sometimes give them some juice by mouth for their enjoyment.

Similar to those with no motor function, this child experienced tiredness and fatigue.

*“He feels tired all the time. He seems like he just kind of wants to lay there, it doesn’t seem like he really wants to do much"* – 2 years (participant 605)

The caregiver also said the individual had trouble sleeping and that they gave them melatonin to help with this. They also used mechanical ventilation at night due to sleep apnoea.

#### Impact on the individual with AADC deficiency

Similar to those with no motor function, the caregiver described this individual as being severely limited in their daily activities because they were unable to move around independently or make functional hand movements. At the time of the interview, the individual was not old enough to attend school and did not attend nursery.

Because the individual was unable to move around, they were unable to play with other children. As a result, their main leisure activities included watching television and being read to by their caregiver.

*“He has trouble moving around...when we do have play time, I’m… we’re kind of sitting on the floor or laying down on the floor and reading him a book or because he really can’t do very much”* – 2 years (participant 605)

Similar to those with no motor function, the caregiver reported that this individual would become upset and frustrated when they were unable to do something.

*“I think he’s sad sometimes. I think he wants to do a lot of things… he wants to be moving around, he wants to be playing, but he can’t, like he tries to lift up his arms and tries to lift up his neck, while we’re watching TV, he tries to, like, move towards the TV and he can’t, so he gets very frustrated”* – 2 years (participant 605)

#### Impact on caregivers

The caring responsibilities of this caregiver were similar to those of individuals with no motor function. The caregiver described how they needed to help the individual with all aspects of their care. They also described how they had to manage their medical routine, including cleaning their feeding tube and giving them their medication.

The caregiver reported that their caring responsibilities had an impact on their physical health, including back pain as a result of lifting and carrying. They also reported being tired because of lack of sleep. This individual did not discuss the emotional impact of caring in a lot of detail, but they did describe a sadness they felt at knowing that the individual would not have the life they wanted for them.

*“It does make me sad because, you know, he’s two and he should be out there running around…climbing on things, he should be jumping off the couch, jumping off the bed, like what two year old’s do”* – 2 years (participant 605)

The caregiver reported that they had needed to stop working in order to become a full-time caregiver. They also described how they were less able to be social than they used to be and struggled to go out with friends because of their caring responsibilities.

### Symptoms and impacts among those able to stand/step (Fig. 3)

**Fig. 3:**
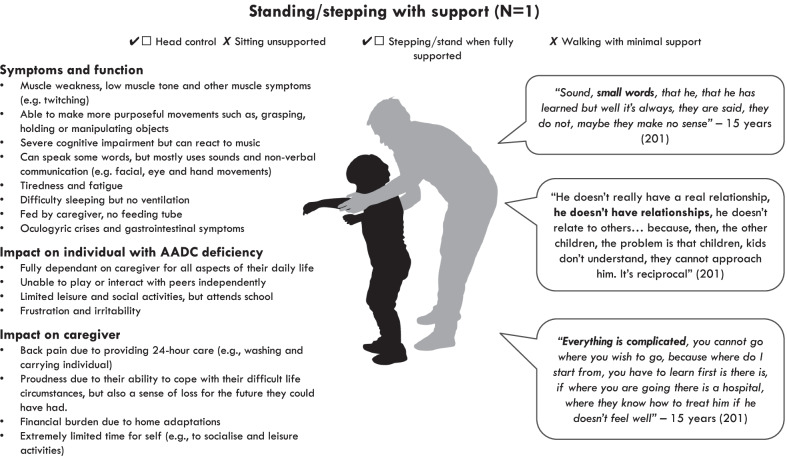
Overview of symptoms and impacts among those able to stand/step

#### Symptoms and function

The one individual in the ‘standing/stepping’ health state was able to hold their head up and could stand/take steps with support, but they were unable to sit unsupported for 60 seconds or walk. Their inability to sit was somewhat surprising as typically individuals would be expected to be able to do this if they could stand, but the qualitative data was taken at face value and the clinician categorised the individual accordingly.

In contrast to those in the ‘no motor function’ and ‘sitting’ health states, this individual was reported to be able to use their hands to grasp, hold or manipulate objects, as well as reach for objects. They were reported to experience other muscle and joint symptoms, including muscle spasms, twitches, and stiffness.

This caregiver also reported that the individual had some limitations with cognitive function, including memory. However, they also reported that the individual was aware of their surroundings and would react to sounds and faces.

*“When he hears music he reacts, yes to music”* – 15 years (participant 201)

In contrast to those in the ‘no motor function’ and ‘sitting’ health states, this individual was reported to be able to communicate using small words, but they could not speak in full sentences and the caregivers said the words did not always make sense. This individual was also reported to use non-verbal communication, including facial expressions, eye movements and hand movements.

The caregiver described episodes that resembled oculogyric crises, but it was unclear if they may have been talking about epileptic seizures, as this was something they had also experienced in the past*.* This caregiver also reported that the individual experienced some gastrointestinal issues, including diarrhoea, reflux and vomiting. The child did not have a feeding tube and was fed blended food by their caregiver, who described how they would sometimes choke when drinking liquids.

*“We have to use thickening agents… it’s not really solid, solid but with the thickening agents it’s better, the fluid one, he chokes”* – 15 years (participant 201)

This caregiver also reported that the individual experienced tiredness and fatigue as well as trouble sleeping at night. They did not use any assisted ventilation devices at night or during the day.

#### Impact on the individual with AADC deficiency

The caregiver described the individual’s daily activities to be limited and that the individual was fully dependent on them for their daily routine and school.

*“He cannot, it’s not that he can do major things, in the sense a typical day, we wake up, I wash him, I prepare, I give him his medicines, I give him breakfast, we see if we have to do some visits, I take him and then I take him to the private facility*" – 15 years (participant 201)

Similar to the ‘no motor function’ and ‘sitting’ health states, their main leisure activities were reported to be watching television, although the caregiver also mentioned that they sometimes try and do something creative together. Although this individual was able make functional hand movements, their limitations with motor function made it difficult for them to make friends as other children did not understand their condition.

The caregiver also reported that the individual was sometimes irritable and nervous when they wanted something or were unable to do something.

*“If he wants to do something, he wants to hold a car that is a little bit bigger in his hand and he cannot do it, he becomes irritable, he becomes nervous and he pushes it away*” – 15 years (participant 201)

#### Impact on caregivers

The caring responsibilities of this caregiver were similar to those caring for individuals in the ‘no motor function’ and ‘sitting’ health states. They described how they needed to help the individual with all aspects of their care, although they did not have any feeding tubes to clean. They described how they needed to plan everything which made it difficult for them to go away and travel with the individual.

The caregiver reported fewer physical impacts than those of caregivers of individuals in the ‘no motor function’ and ‘sitting’ health states. Although they mentioned a small about of pain from carrying the individual, but they did not consider this to be problem. They also did not report any tiredness or fatigue. However, they did report a substantial emotional impact of being a caregiver, including depressive symptoms and grieving a loss of the future they wanted.

*“It’s very difficult, emotionally it’s very heavy, psychologically heavy, and what else can I say, and then my life as well, I don’t want to be misinterpreted, because in a way, my life has changed, my life it’s not the life I wanted to have with my son”* – 15 years (participant 201)

This caregiver also reported an impact on their social life as they were unable to leave the individual to go out with friends or travel away from home with their child. This caregiver had their own business, so their work was not impacted.

### Symptoms and impacts among those able to walk with minimal support (Fig. 4)

**Fig. 4:**
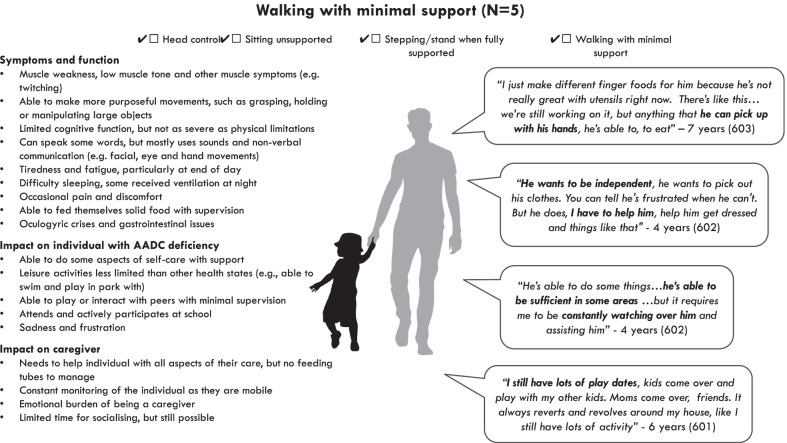
Overview of symptoms and impacts among those able to walk

#### Symptoms and function

In line with their health state categorisation, those in the ‘walking’ health state were able to hold their head upright, sit unsupported, stand and walk with minimal support. Despite being able to walk, those in this health state still had limited motor function relative to healthy individuals, and could only walk short distances, needed to walk more slowly than healthy individuals and needed to take rests.

*“He probably could take a good, 10 foot step, maybe with no support, going very slow”* - 6 years (participant 601)

Some caregivers also reported that individuals in this health state still used a wheelchair or pushchair to help them get around and to give them a rest from walking.

Similar to those in the ‘standing/stepping’ health state, but in contrast to those in the ‘no motor function’ and ‘sitting’ health states, those in this health state were able to make more purposeful movements such as grasping, holding and manipulating objects with their hands. One caregiver reported that the individual was typically able to do this with larger objects, but would struggle with smaller objects.

*“Anything that is… is too small, you know, she will have a problem with grasping it…as far as toys and stuff like that, puzzle pieces, different things that she interacts with, they are larger and easier to grasp"* – 4 years (participant 606)

Another caregiver described how the individual’s functional hand use would vary from day to day depending on their energy levels. Some individuals in this health state were able to raise their hands above their head, whereas others were not. Similar to the other health states, these limitations with motor function were attributed to muscle weakness and low muscle tone.

*“He doesn't have the tone. He gets muscle spasms and there's muscle weakness. So, it's more, everything is in small increments, a lot of rest is needed" -* 6 years (participant 601)

Some caregivers also reported other muscle and joint symptoms, including muscle spasms, twitches, stiffness and abnormal/rigid posture.

Caregivers reported that individuals in this health state had limited cognitive function, although these were described as less severe than the other health states and not as severe as their physical limitations.

*“I think there’s more understanding from a cognitive standpoint, where there’s not as many delays cognitively as there is from the physical aspect” –* 1 year (participant 607)

They described how individuals had an awareness and understanding of their surroundings and were able to recognise faces and remember people and objects. Similar to those in the ‘standing/stepping’ health state, some caregivers reported that the individuals were able to use words to communicate, although they were unable to speak in full sentences.

*“Baby words, nothing significant, not like having a full conversation” -* 6 years (participant 601)

They were also reported to use non-verbal communication, including eye contact, facial expressions and hand movements.

Similar to the other health states, individuals in this health state were reported to experience episodes of oculogyric crises. There were no differences in how these were described compared to other health states. Some individuals were also reported to experience epileptic seizures, particularly at night, but were on medication to control these.

Caregivers reported that individuals experienced a range of gastrointestinal issues, including diarrhoea, constipation, reflux, vomiting and stomach pain. There was some evidence that these gastrointestinal issues were more commonly reported in this health state compared with the ‘no motor function’ health state. This may be attributed to some individuals in the ‘no motor function’ health state having a feeding tube which was used to manage these symptoms. No individuals in this health state had a feeding tube, all ate by mouth. Several caregivers reported that the individuals were able to feed themselves solid foods using their hands.

Similar to the other health states, caregivers reported that individuals in this health state experienced tiredness and fatigue, particularly a night after a day of activity.

*“It’s at night, when he starts to get super tired and he… I don’t want to say he loses the capability of doing those things, but you can just tell that he’s tired and he needs more help”* – 7 years (participant 603)

They also reported sleeping difficulties and some gave the individuals melatonin to help with this. One individual in this health state received assisted ventilation to help them breath at night.

Some caregivers reported that the individuals experienced pain in the stomach and others reported muscle pain.

#### Impact on the individual with AADC deficiency

Caregivers of individuals in the ‘walking’ health state reported that the individuals had more independence than those in the other health states with regard to self-care. For example, some described how the individual was able to select their own clothes, dressing and brushing their teeth with assistance. Caregivers also reported that individuals in this health state participated in a wider range daily activities than the other health states, for example, using crayons, playing with animals, playing in the park and swimming. However, they were still limited in these activities and needed support from their caregiver.

*“I’ll put her up on the slide and she really enjoys, you know, being in the swimming pool… I’d say that in some ways… she’s up there with her age range, but from a physical aspect she is, you know, definitely behind and needs more assistance”* – 4 years (participant 606)

Some individuals in this health state attended school or nursery, whereas others did not, although in some cases this was due to the COVID-19 restrictions. Those who attended school were reported to have specialist support from a teaching assistant or social worker. School was generally described as a positive experience which the individual enjoyed.

*“He loves going to school. It’s only two days a week and he likes to participate with… I mean, mostly he’s with his teacher and the teacher’s aide…there’s other kids in there as well, but they’re, they have other disabilities, but yeah, he enjoys going to school”* – 7 years (participant 603)

Caregivers of individuals in this health state described how their social interaction was limited because other children did not understand their condition. As a result, several caregivers reported that the individual mainly played with their siblings or cousins.

*“We have gone to parks and stuff and it’s a little bit more frustrating with some of the little kids that don’t understand what he’s going through, and you’re at a park, so you’re not gonna, you know, say, “Hey, yeah. My child’s different.” So he’ll play with his cousins primarily”* – 7 years (participant 603)

Similar to those in the other health states, caregivers described how individuals would become upset and frustrated when they were unable to communicate or express themselves.

#### Impact on caregivers

Caregivers of individuals in the ‘walking’ health state described similar caring responsibilities to the other health states, for example, helping the individual with all aspects of their self-care. In addition to the individual’s medical routine taking up a lot of time, caregivers described how they needed to monitor the individual more frequently because they were more mobile. This meant that they had little time to themselves to take part in leisure or social activities. They also described how they needed to plan and schedule everything and were therefore unable to be spontaneous.

*“It’s a big commitment and it’s a lot and you do need to sacrifice a lot. Free time, socialisation, going out and doing things…I would is say is like the biggest impact has been that lack of spontaneity and having to have a schedule and not being able just to go up and take off and do things without, you know, zero planning”* – 4 years (participant 606)

However, some caregivers reported that they were able to have a social life with the individual, particularly if it revolved around their own home. For example, some reported having play dates with other children, which as less commonly reported in the other health states.

From a physical health perspective, fewer caregivers reported experiencing back pain from lifting and carrying the individual, and there were fewer reports of sleeping difficulties. However, some caregivers did report some pain and others described feeling tired and fatigued from their constant caring responsibilities.

*“Time, energy, never being able to be away, or rest. Having to mentally be aware as well as physically” -* 4 years (participant 602)

Similar to the other health states, some caregivers reported depressive symptoms and feelings of sadness. Others described feelings of anxiety and worry about the future.

*“The negatives, of course, you don’t want to see your child have to struggle…there’s been times where I have been super depressed”* – 7 years (participant 603)

## Discussion

This is the first qualitative study to describe the lived experience of individuals with AADC deficiency in different health states, as well as the experience of caregivers. The results highlight a substantial impact of AADC deficiency, even among those who were able to walk with minimal support. In general, those with better motor function also had better functional hand use, communication skills, ability to eat and perform other activities independently, and interact with their peers. This was most apparent for those in the walking health state, who had greater independence that those in the other health states. However, these differences were not all apparent across every health state. The burden of caring was high across all health states, but there was some evidence that caregivers of individuals in the walking health state were better able to participate in social and leisure activities.

Our results showed that while the types of symptoms and functional impairment due to AADC deficiency remained broadly similar across the different health states, there was variation in the level of impairment and the subsequent impact on daily activities. There were fewest notable differences between those with no motor function and those who were able to sit unsupported. In both groups, individuals were reported as being very limited both physically and cognitively. The only notable difference in symptoms was that pain was more commonly reported among those with no motor function, which was attributed to lack of movement, which aligns with the health state definitions. The high level of impairment in both of these health states meant that individuals had very little ability to engage in daily or social activities. As a result, they were fully dependent on their caregivers for all aspects of their lives. This meant that the caregiver burden was high in both groups, with caregivers reporting that they had very little time for themselves, had sometimes needed to quit or change jobs, and had limited opportunities for leisure and social activities. There were some small differences in the emotional impact reported across the two health states, with caregivers of individuals with no motor function reporting more worry and anxiety, and the caregiver of the individual in the sitting health state reporting more sadness. However, it is difficult to draw any conclusions as there was only one individual in the sitting health state.

More notable differences were seen when comparing those with no motor function and those able to sit, with those who were able to stand/step and walk. In contrast to those in the no motor function and sitting health states, those in the stepping/standing and walking health states were able to grasp, hold and manipulate objects. Not only did this mean they were better able to play with toys, but it also aided their non-verbal communication as they were able to communicate with hand gestures. Individuals who could step/stand and walk were also reported to be able to speak a few words, which further improved their communication skills. Also of note is that no individuals in the standing/stepping and walking health states were fed through a feeding tube and could therefore eat by mouth. In addition, those in the walking health state were reported to be able to feed themselves solid foods with their hands. In terms of daily activities, those in the walking health state were reported to have more independence with regard to self-care and were better able to participate in leisure activities and interact with their peers than those in the other health states.

There were also some important differences between health states in terms of the impact on caregivers. Caregivers of individuals who were able to walk reported being slightly better able to participate in social and leisure activities, although they noted that they would need to plan these in advance and were unable to be spontaneous. In some cases improvements for the individual did not necessarily translate to an improvement for the caregiver. For example, while it was perceived as a positive that individuals in the standing/stepping and walking health states could eat by mouth or feed themselves, there was a downside as it took longer for the individual to eat and get all the nutrients they needed. In addition, while it was perceived as a positive for the individual to be able to walk with minimal support, as they were still very limited, the caregivers reported needing to constantly monitor them, which was an added time burden.

This study reports novel information on the lived experience of individuals AADC deficiency and their caregivers across different health states defined by the PDMS-2. Although previous studies have reported utility weights associated with these health states [13], it can be difficult to interpret what this means in real-life terms for individuals and their caregivers. This study provides a narrative around what it means to be an individual in with AADC deficiency or a caregiver in each health state. This is particularly important in rare diseases, such as AADC deficiency, where there is very little known about the disease and how it impacts individuals and caregivers. These findings can help inform HTA agencies on the burden of disease in AADC deficiency and the importance of new treatments which improve motor function.

While this study provides novel insights, it also has a number of limitations that need to be acknowledged. Due to the very small population of individuals living with AADC deficiency, no sample quotas were set for each health state, which resulted in no individuals in the ‘head control’ health state and only one in the ‘sitting’ and ‘standing/stepping’ health states. Although qualitative research is not designed to be representative, a more balanced sample of individuals in the different health states may have yielded additional information. Similarly, the sample were predominantly recruited from the US and Italy, were White and university educated, so the findings may vary in and may not be transferable to other populations. However, as AADC deficiency is an extremely rare disease, recruitment relies on a limited pool of individuals agreeing to take part. Diagnosis of AADC deficiency was caregiver-reported and participants were screened using pre-defined inclusion/exclusion criteria, but this method is not as robust as clinician-confirmed diagnosis. For convenience, particularly during the COVID-19 pandemic, all interviews were conducted by telephone. While this enabled us to speak to caregivers across the globe, non-verbal cues can be missed with telephone interviews and it can be harder to build rapport with participants.

Despite these limitations, this study provides novel insights into the experience of individuals with AADC deficiency and their caregivers across different health states. These insights are from the first qualitative study conducted with caregivers of individuals with AADC deficiency and include data from participants from multiple countries. Given the rarity of the disease, the sample size is large for a qualitative study, and in some cases includes all caregivers of individuals with AADC deficiency in the country.

## Conclusions

Individuals with AADC deficiency with higher levels of motor function also had less severe symptoms and were better able to perform their daily, leisure and social activities. Treatments which improve motor function have the potential to improve other aspects of the lives of individuals with AADC deficiency and their caregivers.

## Data Availability

The datasets used and/or analysed during the current study are available from the corresponding author on reasonable request.
